# Systematic Culturomics Shows that Half of Chicken Caecal Microbiota Members can be Grown in Vitro Except for Two Lineages of *Clostridiales* and a Single Lineage of *Bacteroidetes*

**DOI:** 10.3390/microorganisms7110496

**Published:** 2019-10-28

**Authors:** Magdalena Crhanova, Daniela Karasova, Helena Juricova, Jitka Matiasovicova, Eva Jahodarova, Tereza Kubasova, Zuzana Seidlerova, Alois Cizek, Ivan Rychlik

**Affiliations:** 1 Veterinary Research Institute, 621 00 Brno, Czech Republic; crhanova@vri.cz (M.C.); karasova@vri.cz (D.K.); juricova@vri.cz (H.J.); matiasovicova@vri.cz (J.M.); jahodarova@vri.cz (E.J.); kubasova@vri.cz (T.K.); seidlerova@vri.cz (Z.S.); 2 Central European Institute of Technology (CEITEC), University of Veterinary and Pharmaceutical Sciences Brno, 612 42 Brno, Czech Republic; 3 Department of Infectious Diseases and Microbiology, Faculty of Veterinary Medicine, University of Veterinary and Pharmaceutical Sciences Brno, 612 42 Brno, Czech Republic

**Keywords:** chicken microbiota, microbiome, caecum, culturomics, anaerobic culture, selective culture

## Abstract

Epidemiological data show that the composition of gut microbiota influences host health, disease status, and even behaviour. However, to confirm these epidemiological observations in controlled experiments, pure cultures of gut anaerobes must be obtained. Since the culture of gut anaerobes is not a simple task due to the large number of bacterial species colonising the intestinal tract, in this study we inoculated 174 different culture media with caecal content from adult hens, and compared the microbiota composition in the original caecal samples and in bacterial masses growing in vitro by 16S rRNA sequencing. In total, 42% of gut microbiota members could be grown in vitro and since there were some species which were not cultured but for which the culture conditions are known, it is likely that more than half of chicken gut microbiota can be grown in vitro. However, there were two lineages of *Clostridiales* and a single lineage of *Bacteroidetes* which were common in chicken caecal microbiota but resistant to culture. Of the most selective culture conditions, nutrient broths supplemented with mono- or di-saccharides, including those present in fruits, positively selected for *Lactobacillaceae*. The addition of bile salts selected for *Veillonellaceae* and YCFA (yeast casitone fatty acid agar) enriched for *Desulfovibrionaceae*. In addition, *Erysipelotrichaceae* were positively selected by colistin, trimethoprim, streptomycin and nalidixic acid. Culture conditions tested in this study can be used for the selective enrichment of desired bacterial species but also point towards the specific functions of individual gut microbiota members.

## 1. Introduction

Assessing gut microbiota composition is currently relatively simple due to recent developments in DNA sequencing techniques. Consequently, various studies have associated the composition of gut microbiota with age, disease or behaviour [1,2,3,4]. However, the vast majority of published data are based on epidemiological studies and correlation analyses, which may not necessarily prove the causative link between the tested intervention, microbiota composition and host response. In addition, the correlation analysis cannot distinguish between the cause and the consequence—is the change in gut microbiota composition the cause of the behavioural change or its consequence?

Studies administering defined bacteria, followed by verification of their presence in the intestinal tract and their association with tested characteristics, are much less frequent and most frequently involve the administration of *Lactobacillaceae* family members [5,6,7,8,9]. However, *Lactobacillaceae*, though common in the small intestine, form only a small fraction of gut microbiota in the distal parts of the intestinal tract like the caecum [10], microbiota of which is formed by hundreds of anaerobic bacterial species different from *Lactobacilli* [1,5]. Because of this, we and others have initiated systematic culturing of gut anaerobes [11,12,13,14] to have them available for whole genome sequencing and subsequent experiments.

Soon after we formed an initial laboratory collection of chicken gut anaerobes, we compared the isolates from the collection with those commonly found in chicken intestinal tract. In other words, we were interested whether there were any chicken gut anaerobes which were difficult to culture. Other studies addressed the same question by comparing the bacterial composition in original faecal or caecal samples with those in nutrient broth after culture [12,13,14]. These studies concluded that between 50 to 90% of total gut microbiota could be cultured in vitro if the appropriate culture conditions are provided [13,14]. Unfortunately, these studies did not perform phylogenetic analyses of the OTUs (operational taxonomic units) capable or incapable of in vitro growth. Therefore, it is not known whether the bacterial species resistant to in vitro growth are randomly distributed among known bacterial phyla, classes, orders, families, genera or species, or whether the microbiota members resistant to in vitro growth are clustered in particular lineages, which is exactly what we investigated in this study. Using a larger set of different culture conditions, the primary aim was to determine which bacterial lineages colonising the chicken caecum have not yet been cultured in vitro. Furthermore, the experimental design also enabled us to address secondary aims such as to which extent feed or food additives, therapeutics or drugs influence in vitro bacterial growth in complex microbial populations and what are the most selective culture conditions for particular taxa. Indirectly, such data also points towards possible functions of individual microbiota members in vivo.

## 2. Materials and Methods 

### 2.1. Samples for Analysis

The ability of in vitro bacterial growth was tested in six independent experiments. In each experiment, the whole caecum with its contents was collected from ISA Brown hens (egg laying line) 34–40 weeks of age and transferred to an anaerobic cabinet within 30 min. The caeca were opened in the cabinet, approx. 0.5 g of the content was resuspended in 5 mL of pre-reduced PBS (phosphate buffered saline), 10-fold serially diluted and multiple dilutions were used for inoculation of nutrient broths or plated on nutrient agars (see Table 1 for a brief overview and Table S1 for a detailed list of tested culture media). In the first experiment, 60 different culture conditions were tested. In the second experiment, 12 conditions; in the third experiment, 14 conditions; in the fourth experiment 40 conditions, in the fifth, experiment 33 conditions; and in the last experiment, 15 conditions were tested so that the caecal contents were plated on 174 differently supplemented media in total. Inoculated media were incubated for 48 h in an anaerobic atmosphere at 37 °C as described previously [11]. Following the growth in liquid media, the bacterial mass was obtained by centrifugation for 1 min at 14,000 × g. In the case of nutrient agars, the bacterial mass was obtained by washing all growing colonies from the surface with 5 mL of PBS followed by pelleting the bacterial mass by centrifugation for 1 min at 14,000 × g. Bacterial DNA was purified from the pellets and from the original caecal contents by QIAamp DNA Stool Mini kit (Qiagen, Hilden, Germany). Next, the DNA was used as a template in PCR with eubacterial primers amplifying the V3/V4 variable region of 16S rRNA genes, and the microbial composition in the caecal samples and in each culture media was determined by 16S rRNA sequencing exactly as reported previously [5].

### 2.2. OTU Selection and Definition of In Vitro Growth

Sequencing data from all six experiments were processed together using a single Qiime pipeline [15]. PCoA plots used for diversity presentation were calculated using data for all OTUs. However, the analysis of bacterial species which grew or did not grow in vitro was limited to the OTUs which formed more than 0.1% of total microbiota in at least one of the six caecal samples. This threshold was passed by 341 OTUs which were subsequently checked for their ability to grow in vitro. Since the bacterial mass was washed from plates containing approx. 1000 growing colonies, and the bacterial mass from liquid media was collected from tubes inoculated from the dilution which resulted in approx. 1000 growing colonies on nutrient agars, the threshold for an OTU being considered as growing in vitro was set to 0.1% of the total microbial community.

### 2.3. 16S rRNA Alignment

All partial 16S rRNA sequences were aligned by Mafft with Q-INS-I algorithm [16]. To obtain a more precise view of bacteria growing in vitro, 16S rRNA sequences of 307 gut anaerobes which we already had in pure cultures [11], i.e., which can grow in vitro, were included in this analysis as well. trimAl 1.3 was used to remove ambiguous positions in alignment with -gt parameter set to 0.25 [17]. Bayesian inference was estimated using BEAST 1.8.1 [18] with the HKY+I+G model, applying a strict clock (rate = 1). The analysis was run for 150,000,000 generations, with 10,000 sampling frequency and Saccharibacteria genera incertae sedis c202 used as an outgroup. Tracer v.1.7 (https://github.com/beast-dev/tracer/releases/latest) was used for checking ESS values and running convergence plateau [19], and a maximum credibility tree was estimated using TreeAnnotator after discarding the initial 30% of trees [18]. Maximum likelihood analysis was performed in IQ-TREE [20] and the substitution model was identified using ModelFinder (http://www.iqtree.org) implemented in IQ-TREE (option -m TEST) [21]. Maximum likelihood analysis was tested using ultrafast bootstrapping with 10,000 replicates [22].

### 2.4. Ethics Statement

The handling of animals in the study was performed in accordance with current Czech legislation (Animal Protection and Welfare Act No. 246/1992 Coll. of the Government of the Czech Republic). The specific experiments were approved by the Ethics Committee of the Veterinary Research Institute followed by the Committee for Animal Welfare of the Ministry of Agriculture of the Czech Republic (permit number MZe1922).

## 3. Results

### 3.1. Identification of OTUs Common to the Chicken Caecum and Their Growth In Vitro

In total, 10,938,758 reads were analysed in this study. Median and mean sequence coverage were 52,419 and 60,770 reads per sample, respectively. Sample coverage ranged from 15,481 reads in the samples with the lowest coverage to 261,333 reads in the sample with the highest coverage (Table S2). Altogether, 200,551 OTUs were identified of which 341 OTUs were present in the caecal contents of at least one of the donor hens at ≥0.1% abundance. Of these, 144 OTUs (42.23%) reached ≥0.1% abundance in at least one of the tested nutrient broths, i.e., these OTUs grew in vitro. At the phylum level, we failed to culture OTUs belonging to *Spirochaetes*, *Deferribacteres*, *Elusimicrobia*, *Verrucomicrobia*, *Candidatus Saccharibacteria* and *Tenericutes* though each of these phyla were represented by three or less OTUs. On the other hand, OTUs belonging to *Fusobacteria* and *Actinobacteria* grew easily in vitro although representatives of these phyla did not belong among the most frequent microbiota members in the samples tested in this study (Table 2). Of the most common chicken gut microbiota members belonging to phyla *Bacteroidetes*, *Firmicutes* and *Proteobacteria*, 20 OTUs belonging to *Proteobacteria* did not grow in vitro, and this phylum therefore comprised the highest proportion of in vitro non-growing OTUs (62.5%). Of the OTUs belonging to phyla *Firmicutes* and *Bacteroidetes*, 46.06% belonging to *Firmicutes* and 42.29% of *Bacteroidetes* could be grown in vitro (Table 2).

**Figure 1:**
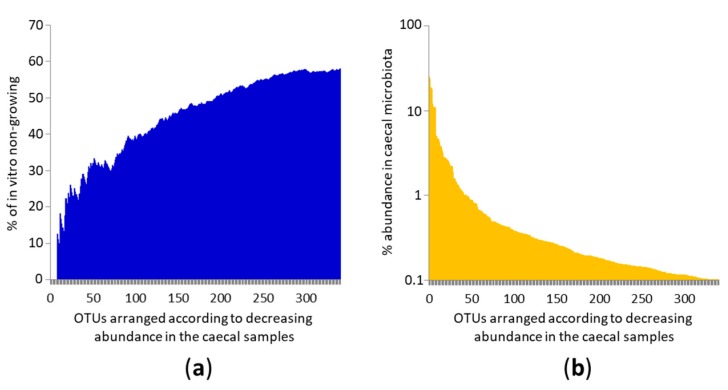
Ability of in vitro growth of the most frequent OTUs colonising the chicken caecum. (**a**). Individual OTUs are arranged on the X axis according to their decreasing abundance in chicken gut microbiota. Y axis shows the proportion of non-growing OTUs. The most common OTUs grew in vitro and their ability to grow in vitro decreased as OTUs became less common. (**b**) X axis shows the same values as in panel (**a**) and the Y axis shows the abundance of a given OTU in the caecal microbiota. Each of the top 45 OTUs formed more than 1% of total microbiota in at least one of six caecal samples used as inocula for in vitro culture. Mind the logarithmic scaling of the Y axis in panel (**b**).

Although only 42.23% of the analysed OTUs grew in vitro, the distribution of growing and non-growing OTUs among the top 341 OTUs from chicken gut microbiota was not linear. Of the top 10 OTUs, we failed with culture of only a single OTU identified as *Helicobacter pullorum*. Of the top 50 OTUs, 66.66% of OTUs could be cultured and of the top 100 most common OTUs in the caecal sample, we could still culture 61% of them (Figure 1). This means the likelihood of being cultured decreased with decreasing OTU abundance in the original caecal sample.

**Figure 2:**
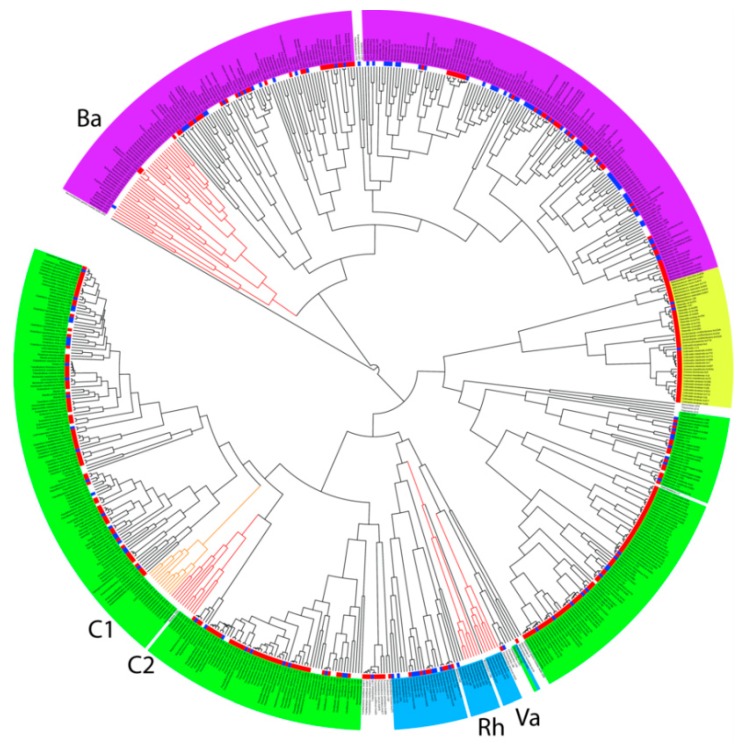
Taxonomic classification of the most common bacterial isolates colonising the chicken caecum and their ability to grow in vitro. Magenta background—*Bacteroidetes*, blue—*Proteobacteria*, yellow—*Actinobacteria*, and green—*Firmicutes*. Blue squares external to the dendrogram indicate the ability to grow in vitro using different culture conditions. Red squares indicate isolates which we obtained in pure cultures in our previous experiments, i.e., which can be grown in vitro. Lineages difficult to culture in vitro are highlighted by red or orange branches. Ba—*Bacteroidetes* lineage, C1 and C2—two different lineages of *Clostridiales*, Rh—*Rhodospirillaceae*, Va—*Vampirivibrio*. See Figure S1 to zoom in.

Clustering of the most abundant OTUs in the chicken caecum according to their partial 16S rRNA sequence and their ability to grow in vitro is shown in Figure 2. OTUs belonging to *Actinobacteria* were simple to culture since OTUs belonging to genera *Bifidobacterium*, *Collinsella*, *Olsenella* of *Gordonibacter* grew in vitro. Among *Proteobacteria*, isolates from *Enterobacteriaceae*, *Sutterrellaceae*, *Desulfovibrio*, *Succinatomonas* and *Anaerobiospirillum* could be grown in vitro (Figure 2). On the other hand, media and culture conditions tested in this study did not support the growth of *Rhodospirillaceae*, *Vampirivibrio*, *Helicobacter* and *Campylobacter*.

Among *Firmicutes*, isolates from orders *Bacilli* and *Selenomonadales* belonged among the taxa simple to culture. Isolates from three main families belonging to order *Clostridiales*, i.e., *Lachnospiraceae*, *Ruminococcaceae* and *Erysipelotrichaceae*, were also rather simple to culture. However, there were two lineages of *Clostridiales* which were common to the chicken caecum but resistant to culture (Figure 2). Each of these lineages comprised 11 OTUs which were only classified down to order *Clostridiales*. Two new families within the order *Clostridiales* therefore remain to be cultured and identified in detail. Most of the OTUs belonging to *Bacteroidetes* could be cultured in vitro, except for a single lineage of isolates which were difficult to culture (Figure 2). This lineage comprised 32 OTUs and 19 of them were only annotated down to phylum level.

### 3.2. Specific Analysis of 16S rRNA Sequences

Partial 16S rRNA sequences of all OTUs belonging to the lineages resistant to culture were manually BLAST compared against GenBank entries, using either 16S ribosomal RNA sequences (Bacteria and Archaea) database or nucleotide collection (nr/nt) database. When using the 16S ribosomal RNA database, similarities of any of the 66 OTUs belonging to C1, C2, Rh, Va and Ba lineages to the closest database entry never exceeded 90%. When blasting OTU sequences belonging to C1, C2, Rh and Va lineages against non-redundant database, similarities increased to 95–100%. This means that these OTUs were earlier independently recorded by other authors as uncultured bacteria and represent real bacteria requiring unknown growth conditions. For the Ba *Bacteroidetes* lineage, only seven OTUs were similar to nr/nt database entries by more than 95% and similarities for the remaining OTUs ranged from 84.31 to 94.58%. To exclude that these OTUs represented chimeric sequences (though chimera identification and removal was included in the Qiime pipeline), we separately BLAST compared 80 nt from the 5′ and 3′ ends of all OTUs belonging to Ba lineage. Only a single OTU likely represented a chimera which escaped from chimera removal step since the 5′ end sequence of this OTU was 100% identical to *Bacteroides gallinaceum* and the 3′ end sequence of the same OTU was 86.59% similar to *Williamwhitmania taraxaci*. Since both ends of the sequences of the remaining unculturable *Bacteroidetes* OTUs were around 90% similar to nr/nt database entries, we excluded the chimera hypothesis and concluded that these OTUs represented real but not yet cultured *Bacteroidetes* OTUs. Unlike C1 and C2 lineages of *Clostridiales*, *Rhodospirillaceae* and *Vampirivibrio*, the Ba lineage OTUs might be strictly chicken-adapted and therefore less commonly deposited in the GenBank database.

### 3.3. Culture Conditions Selective for Isolation of Particular Taxa

**Figure 3:**
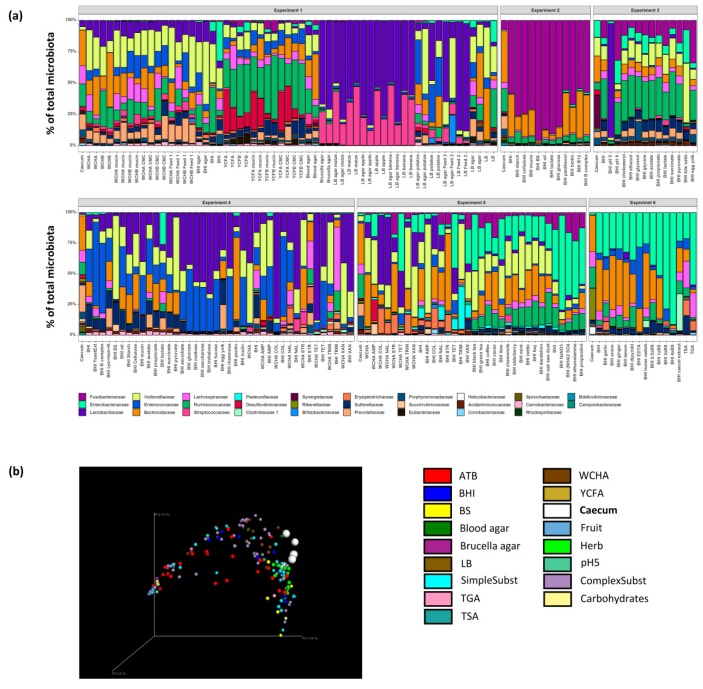
Identification of the most selective growth conditions. (**a**) The six independent experiments differed considerably due to different inoculum and tested growth conditions. (**b**) PCoA analysis of all analysed samples showed that those which differed the most from the original caecal samples originated from media supplemented with bile salts, mono- and di-saccharides, fruits and antibiotics (ATB). See Figure S2 to zoom in.

Variation in the microbiota composition in the caecal samples and variation in the types of media used for inoculation resulted in highly variable outcomes after in vitro culture (Figure 3a, Figure S2 and Table S2). Despite this, PCoA analysis showed that the addition of common herbs, simple substrates (e.g., lactate, pyruvate, ethanol, succinate or glycerol) and complex substrates (e.g., mucin, inulin, cellulose or egg yolk) had a low effect on the positive selection of particular microbiota members. On the other hand, the addition of bile salts to growth media, use of Brucella agar or BHI medium at pH 5, and supplementation of BHI media with fruits, mono- and disaccharides resulted in the most selective growth conditions tested in this study (Figure 3b, Figure S2 and Table S2). The addition of different antibiotics also influenced the composition of growing microbiota (Figure 3b, Figure S2 and Table S2). To identify the most selective growth conditions for particular taxa, we arranged OTUs according to their abundance in different media in descending order and identified the OTUs from different phyla specifically enriched in different broths.

### 3.4. Firmicutes

**Figure 4:**
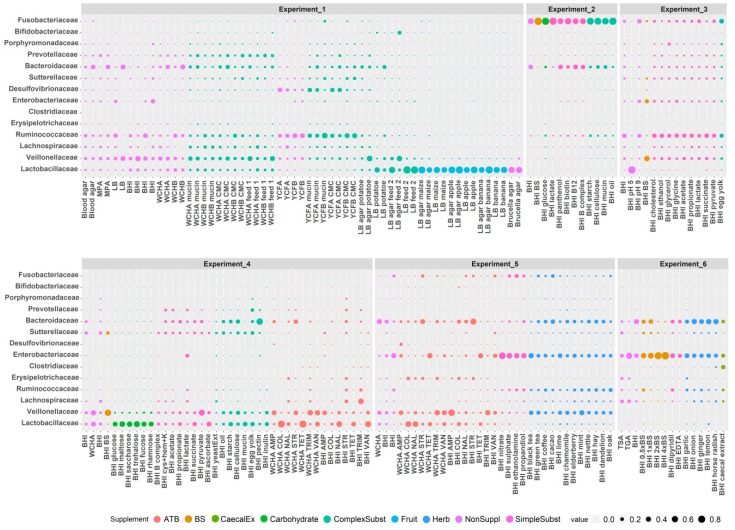
Growth of major families in particular nutrient media in vitro. Individual experiments are separated by a gap. Sizes of the dots indicate the abundance of a given family in a particular growth medium. See Figure S3 to zoom in.

*Ruminococcaceae* grew the best in non-supplemented YCFB and YCFB medium supplemented with mucin or carboxymethyl cellulose. *Ruminococcaceae* were also positively selected in BHI medium supplemented with succinate, ethanol, cholesterol, acetate, propionate, pyruvate and glycine. *Lachnospiraceae* grew the best in non-supplemented TSA, TGA and YCFA. *Lachnospiraceae* were highly represented also in non-supplemented BHI and WCHA, suggesting that none of the supplements tested in this study were specifically selective for *Lachnospiraceae*. *Erysipelotrichaceae* were positively selected in BHI and WCHA supplemented with antibiotics such as colistin, trimethoprim, streptomycin and nalidixic acid. *Erysipelotrichaceae* grew well also in non-supplemented TSA and TGA. *Clostridiaceae* 1 represented by a dominant OTU belonging to *Clostridium perfringens* were enriched under a single growth condition tested in this study—*Clostridiaceae* 1 formed 29.64% of all microbiota in BHI medium supplemented with filter sterilised caecal extract. *Lactobacillaceae* were selected the most after inoculation of caecal contents into BHI medium at pH 5. Additional conditions selecting for *Lactobacillaceae* included supplementation of BHI medium with fruits like apple, banana or sweet maize, disaccharides such as trehalose, maltose or saccharose, feed for chickens, or antibiotics like tetracycline, colistin and vancomycin. The addition of ascorbate into BHI medium also positively selected for *Lactobacillaceae*. *Veillonellaceae* grew well in both liquid and solid media including BHI, WCHA or LB. *Veillonellaceae* were positively selected by bile salts, ampicillin, vancomycin and trimethoprim. *Veillonellaceae* also increased in their abundance after supplementation with complex substrates like potatoes or 2 different types of chicken feed. *Veillonellaceae* were also weakly positively selected by the addition of mint (Figure 4 and Figure S3).

### 3.5. Bacteroidetes

The most selective conditions for *Bacteroidaceae* included the addition of pectin or streptomycin to BHI. However, since the top 15 growth conditions supporting the growth of *Bacteroidaceae* included WCHA, WCHB, BHI, LB and blood agar without any supplementation, *Bacteroidaceae* seems to be able to grow under various conditions *in vitro*. *Prevotellaceae* grew well in BHI, WCHA or WCHB and were enriched after the addition of complex substrates such as egg yolk, chicken feed, carboxymethyl cellulose or mucin. *Porphyromonadaceae* were positively selected in BHI supplemented with egg yolk, glycerol or cholesterol (Figure 4).

### 3.6. Proteobacteria, Actinobacteria and Fusobacteria

*Enterobacteriaceae* were positively selected by bile salts and nitrate. *Sutterellaceae* grew well in TSA and BHI with or without egg yolk, carboxymethylcellulose, sun flower oil, cellulose or mucin. *Desulfovibrionaceae* formed 10–30% of the total microbiota after growth in YCF medium preferring growth on YCFA agar plates to liquid YCFB (Figure 4). *Bifidobacteriaceae* were positively selected only when grown on LB agar supplemented with one of the tested chicken feeds (Figure 4). *Fusobacteriaceae* preferred growth in liquid BHI medium without any specific supplementation (Figure 4).

## 4. Discussion

In this study, we addressed which bacteria of those colonising the chicken intestinal tract can be grown in vitro, and for those growing, what are the most selective conditions which can be used for their culture.

Overall, 42.23% of 341 OTUs present at ≥0.1% abundance in chicken caecal microbiota could be grown in vitro. This number is close to similar studies [13,14]. In fact, the real number of chicken gut microbiota which can be grown in vitro is likely higher since some bacterial genera like *Helicobacter* and *Campylobacter* did not grow under culture conditions which were tested in this study though specific culture conditions for these genera are known [3,23]. Moreover, the ability to culture decreased with the decreasing abundance of the OTU in the initial caecal sample. Lowly abundant OTUs can be disadvantaged in comparison to dominant OTUs from the beginning of in vitro culture by the mere struggle for available nutrients. Future culturing of caecal samples, which may have a higher abundance of the OTUs that were difficult to culture in this study, may allow for their isolation in vitro. This is also a reason why we do not overestimate the biological relevance of the non-growing OTUs which clustered next to the OTUs which grew in vitro. This could just be a matter of coincidence, and if these OTUs were present at a higher abundance in the original caecal samples, we would have cultured them. This was also a reason why we focused more on the non-growing OTUs forming defined lineages.

*Rhodospirillaceae* and *Vampirivibrio* are both known for their specific ecology and although the caecal OTUs were only weakly related to these taxa, it need not be that surprising that we did not meet the conditions required for their growth. However, the two different lineages of *Clostridiales* and one of *Bacteroidetes* belong to phyla characteristic for gut microbiota and therefore should require similar growth conditions as other members of these taxa. The reasons for their inability to grow are currently unknown but OTUs with similar sequences are deposited in the GenBank and therefore must have been observed also by other authors. Additionally, we checked that these OTUs were present in more than one out of six samples used for the inoculations in this study, which means that laboratory artefacts can be excluded. In *Clostridiales*, the two lineages were well-formed, likely representing two new bacterial families. In the *Bacteroidetes* lineage, the distances between individual nodes were higher and future re-classification may include several new families or orders. Interestingly, we cultured two isolates belonging to this lineage previously and characterised them as *Muribaculum*-like species [11]. These two isolates had specific characteristics, encoding genes like 6-aminohexanoate-dimer hydrolase required for nylon degradation and a gene for atrazin chlorohydrolase [11]. Similar unusual characteristics can be expected also for the remaining members of this lineage.

Testing different culture conditions also allowed for understanding the possible roles of these taxa in the gut microbiota community. As expected, *Enterobacteriaceae* were enriched in the presence of bile salts, consistent with the fact that bile salts are present in MacConkey or Endo agars used for *E. coli* culture. However, *Veillonellaceae* were also resistant to bile salts. Since *Veillonellaceae* are usually more abundant in the intestinal tract of adult chickens than *Enterobacteriaceae*, using higher dilutions (to dilute out *Enterobacteriaceae*) and plating on nutrient agars with bile salts will increase the probability of positive culture of *Megamonas* or *Megasphaera* species, both belonging to *Veillonellaceae*. Why *Veillonellaceae* are resistant to bile salts is unclear but this may be associated with the fact that *Veillonellaceae*, though phylogenetically belonging to Gram-positive *Firmicutes*, expresses an outer membrane like Gram-negative bacteria [24]. Bile resistance was expected for *Lactobacilli* which colonise the small intestine rich in bile salts [25]. However, this was not the case. Instead, *Lactobacilli* were resistant to low pH. Rapid metabolism of *Lactobacilli* associated with a decrease in pH due to anaerobic fermentation of carbohydrates into organic acids is the most likely explanation for why *Lactobacilli* dominated in carbohydrate supplemented media.

When growth preferences of the three main families from order *Clostridiales* were compared, they exhibited quite distinct profiles. *Lachnospiraceae* did not show any preference. *Ruminococcaceae* grew the best in YCFB and YCFA, or in complex media supplemented with succinate, ethanol, cholesterol, acetate, propionate or pyruvate. Since acetate and propionate are present also in YCFB as short chain fatty acid supplements, these substrates can be used for enrichment media for *Ruminococcaceae*. *Erysipelotrichaceae*, the last family of *Clostridiales* commonly found in chicken gut microbiota [1], were positively selected in nutrient broths supplemented with antibiotics. Since the selection was reproducible, in different experiments and different media, it is unlikely that this could be caused by horizontally acquired resistances. The more likely explanation is that isolates of this family are intrinsically resistant to antibiotics such as colistin, trimethoprim, streptomycin and nalidixic acid, and these can be used for their enrichment from faecal or caecal samples.

*Clostridiaceae* 1 represented by *Clostridium perfringens* was enriched only in the medium supplemented with caecal extract. The caecal extract originated from the same caecal sample as the inoculum, and *C. perfringens* was not recorded on the remaining plates or broths inoculated with this caecal sample. *C. perfringens* overgrowth therefore could not be associated with high *C. perfringens* counts in the serially diluted sample and instead, must be associated with the addition of caecal extract itself. We considered that bile salts, cholesterol, heme degradation byproducts like bilirubin or billiverdin, or enzymes like trypsin, chymotrypsin, meprin 1A or sucrose isomaltase [26] of host origin supported *C. perfringens* growth. Though such explanations cannot be excluded, we prefer an alternative hypothesis. We propose that *C. perfringens* growth on agar plates was caused by its spores passing through the 0.22 µm filter when preparing the caecal extract. Due to the caecal extract preparation, approx. 10^4^ spores/g of original digesta would result in approx. 30% abundance of *C. perfringens* on agar plates with the caecal extract, which is not an unrealistic estimation for healthy chickens.

Though *Bacteroidaceae* were positively selected by the addition of pectin, this observation was not supported by the enrichment of *Bacteroidaceae* in other media supplemented with complex substrates. It seems that as with *Lachnospiraceae*, *Bacteroidaceae* does not exhibit any positive selection during in vitro culture. Another family belonging to order *Bacteroidales*, *Prevotellaceae*, was enriched after media supplementation with complex substrates. This is in agreement with reports on increased *Prevotellaceae* abundance in humans from rural parts of Africa whose diets were rich in vegetables and fiber [27,28]. *Porphyromonadaceae* were enriched by egg yolk, glycerol or cholesterol. This proposes their role in metabolism of lipids and also may enable them to colonise chickens from the very first days of life when egg yolk still represents a major source of nutrients for the developing chick [29,30].

In this study, we primarily determined which bacterial species and lineages colonising the chicken caecum can or cannot be cultured in vitro. Furthermore, the experimental design enabled us to also address to what extent different feed or food additives, therapeutics and drugs affect bacterial growth in vitro. However, minor modifications of the protocol may allow for alternative experiments. Small aliquots of washed bacterial pools can be frozen at −80 °C before results of 16S rRNA sequencing are available and should the species of interest be enriched under some of the tested culture conditions, the frozen aliquot can be subcultured and the target colony can be sought. If no information on the target species is known, washes from the agar plates can be used for shotgun sequencing followed by metagenomic sequence assembly. Sequences of the desired species can be identified among metagenomic contigs and used for designing specific primers for PCR which can then be used for the identification of the colony of the desired species. Finally, created collection gut anaerobes can be used for oral administration to chickens and preparation of the next generation of probiotics.

## 5. Conclusions

Systematic culture of gut anaerobes, from chickens as well as other animal species including humans, is one of the key ways to better understand the roles of individual microbiota members. In this study, we showed that more than 42% of chicken gut microbiota members could be grown in vitro. The real number of chicken gut microbiota which can be grown in vitro is, however, likely higher since some bacterial species, for which specific culture conditions are known, were classified as non-growing in this study. In addition, there were two lineages of *Clostridiales* and a single lineage of *Bacteroidetes* which were common in chicken caecal microbiota but resistant to culture. Although conclusions of culture experiments will always be influenced by microbiota composition in the original sample and growth conditions (e.g., anaerobic atmosphere and used basal medium), these may deepen the understanding of the behaviour of individual microbiota members.

## Figures and Tables

**Table 1:** List of nutrient media and supplements tested in this study.

Category	Used Nutrient Broths and Tested Supplements
Non supplemented broths	Blood agar, Brucella agar, TGA, BHI, TSA, WCHA, WCHB, YCFB, YCFA, LB agar, LB broth
Complex substrates	caecal extract, carboxymethyl cellulose, hay, mucin, oil, starch, cellulose, egg yolk, feed, inulin, oak saw dust, pectin, potato, yeast extract
Simple substrates	(NH4)_2_ SO_4_, acetate, ascorbate, B complex, vitamine B12, biotin, cystein+hemin+vitamine K, dipyridil, EDTA, ethanol, ethanolamine, glycerol, glycine, cholesterol, lactate, NaNO3, panthenol, propandiol, propionate, pyruvate, succinate
Carbohydrates	glucose, maltose, saccharose, trehalose, fucose, rhamnose
Fruit	apple, banana, maize
Herb	black tea, green tea, coffee, cacao, lime, chamomile, elderberry, mint, nettle, dandelion, garlic, onion, ginger, lemon, horse radish
Antibiotics	ampicillin, colistin, nalidixic acid, streptomycin, tetracycline, trimethoprim, vancomycin
Miscellaneous	bile salts, pH 5, pH 9

**Table 2:** Numbers of OTUs of the most abundant microbiota members in the chicken caecum assigned to different phyla and their ability to grow in vitro.

Phylum	All OTUs	Non-Growing	Growing	% of Growing
Archaea	1	1	0	0
Unclassified Bacteria	2	2	0	0
Candidatus Saccharibacteria	1	1	0	0
Deferribacteres	1	1	0	0
Spirochaetes	3	3	0	0
Elusimicrobia	1	1	0	0
Tenericutes	1	1	0	0
Verrucomicrobia	2	2	0	0
Fusobacteria	1	0	1	100
Synergistetes	3	2	1	33.33
Actinobacteria	3	0	3	100
Bacteroidetes	175	101	74	42.29
Firmicutes	115	62	53	46.09
Proteobacteria	32	20	12	37.5
Total	341	197	144	42.23

## Supplementary Materials

The following are available online at https://www.mdpi.com/2076-2607/7/11/496/s1, Table S1: List of all media tested in this study, Table S2: OTU table with percentage abundance of particular OTU in particular sample, Figure S1: Taxonomic classification of the most common bacterial isolates colonising the chicken caecum and their ability to grow *in vitro*. Figure S2: Identification of the most selective growth conditions. Figure S3: Growth of major families in particular nutrient media *in vitro*.
