# Supplementary figures and images for: Systematic Culturomics Shows that Half of Chicken Caecal Microbiota Members can be Grown in Vitro Except for Two Lineages of Clostridiales and a Single Lineage of Bacteroidetes
Source: Microorganisms. 2019 Oct 28;7(11):496. doi: 10.3390/microorganisms7110496 (PMC6920758; doi:10.3390/microorganisms7110496)

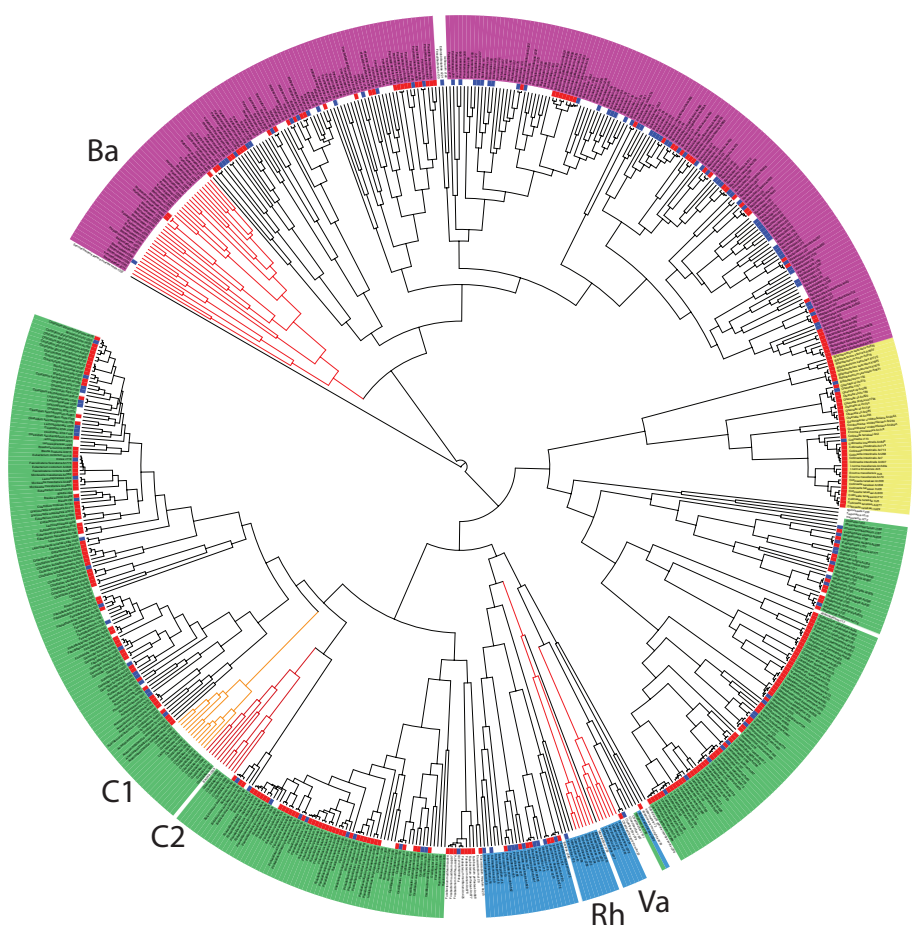

Supplement: Supplementary file 1 [file microorganisms-07-00496-s001.zip › Figure S1.pdf]
